# Capsule endoscopy with flexible spectral imaging color enhancement reduces the bile pigment effect and improves the detectability of small bowel lesions

**DOI:** 10.1186/1471-230X-12-83

**Published:** 2012-07-02

**Authors:** Eiji Sakai, Hiroki Endo, Shingo Kato, Tetsuya Matsuura, Wataru Tomeno, Leo Taniguchi, Takashi Uchiyama, Yasuo Hata, Eiji Yamada, Hidenori Ohkubo, Takuma Higrashi, Kunihiro Hosono, Hirokazu Takahashi, Atsushi Nakajima

**Affiliations:** 1 Gastroenterology Division, Yokohama City University School of Medicine, 3-9 Fuku-ura, Kanazawa-ku, Yokohama, 236-0004, Japan; 2 Gastroenterology Division, Chigasaki City Hospital, Chigasaki, Japan

**Keywords:** Capsule endoscopy (CE), Computed virtual chromoendoscopy system, Flexible spectral imaging color enhancement (FICE), Small bowel

## Abstract

**Background:**

Capsule endoscopy with flexible spectral imaging color enhancement (CE-FICE) has been reported to improve the visualization and detection of small-bowel lesions, however, its clinical usefulness is still not established. Therefore, we conducted a study to evaluate whether CE-FICE contributes to improve the detectability of small-bowel lesions by CE trainees.

**Methods:**

Four gastroenterology trainees without prior CE experience were asked to read and interpret 12 CE videos. Each of the videos was read by conventional visualization method and under three different FICE settings. To evaluate whether the lesion recognition ability of the CE trainees could be improved by the FICE technology, the lesion detection rate under each of the three FICE settings was compared with that by conventional CE. CE trainees tend to miss small-bowel lesions in bile-pigment-positive condition, therefore we evaluated whether CE-FICE contributes to reducing the bile-pigment effect. The bile-pigment condition was determined by the color values around the small-bowel lesions according to the results of the receiver-operating-characteristic analysis. Moreover, we also evaluated whether poor bowel preparion might affect the accuracy of lesion recognition by CE-FICE.

**Results:**

Of a total of 60 angioectasias, CE trainees identified 26 by conventional CE, 40 under FICE setting 1, 38 under FICE setting 2, and 31 under FICE setting 3. Of a total of 82 erosions/ulcerations, CE trainees identified 38 by conventional CE, 62 under FICE setting 1, 60 under FICE setting 2, and 20 under FICE setting 3. Compared with conventional CE, FICE settings 1 and 2 significantly improved the detectability of angioectasia (*P* = 0.0017 and *P* = 0.014, respectively) and erosions/ulcerations (*P* = 0.0012 and *P* = 0.0094, respectively). Although the detectability of small-bowel lesions by conventional CE (*P* = 0.020) and under FICE setting 2 (*P* = 0.0023) was reduced by the presence of bile-pigments, that under FICE setting 1 was not affected (*P* = 0.59). Our results also revealed that in poor bowel visibility conditions, CE-FICE yielded a high rate of false-positive findings.

**Conclusions:**

CE-FICE may reduce the bile-pigment effect and improve the detectability of small-bowel lesions by CE trainees; the reliability of CE-FICE may be improved by good bowel preparation.

## Background

Capsule endoscopy (CE) was introduced in the year 2000 and has since come to be recognized as a useful tool for assessment of the entire small bowel [1-7]. Several studies have reported a higher diagnostic yield of CE as compared to that of other modalities, such as push enteroscopy [8,9], small-bowel radiography [10] and computed tomography [11]; therefore, CE is now established as the examination modality of first choice for the evaluation of small bowel lesions. However, the diagnostic yield of CE may be reduced when the visibility of the mucosa is impaired by the presence of air bubbles, food residues and bile pigments. Although the visibility may be improved by bowel preparation, the benefits of bowel preparation for CE still remain controversial [12-15]. Bile pigments contain bilirubin, look yellow and darken the small bowel, thereby impairing the visibility and lesion recognizability of small bowel lesions, especially by CE trainees. Therefore, it would seem important to devise a method(s) to improve the detectability of small bowel lesions in the presence of bile pigments.

There have been reports of the usefulness of the newly developed computed virtual chromoendoscopy system, namely, flexible spectral imaging color enhancement (FICE), for identifying and diagnosing lesions of the gastrointestinal tract, such as of the esophagus, stomach and large intestine [16-19]. The FICE technology decomposes images by three specific wavelengths (red, green and blue) and then directly reconstructs the images with enhanced surface contrast, thereby improving the visibility of the endoscopic images [19]. The FICE software has recently been incorporated into the new RAPID 6.0 video CE workstation (Given Imaging Ltd, Yoqneam, Israel). With this innovation, the assessor can easily select between conventional images and images reconstructed under three different FICE settings by the click of an icon in the Rapid Reader software for optimal mucosal visualization [20]. Despite the usefulness of CE-FICE, it is not widely used in routine practice, because its clinical usefulness is yet to be firmly established.

FICE has been reported to enhance the visibility of small bowel lesions in CE [21], therefore, we predicted that CE-FICE might also allow improved detectability of lesions under conditions of poor bowel visibility, such as in the presence of bile pigments. First, we attempted to confirm whether CE-FICE improved the detectability of small bowel lesions, such as angioectasia and erosions/ulcerations. Then, we evaluated whether the presence of bile pigments might affect the small bowel lesion detection rate by CE trainees and whether CE-FICE might allow reduction of the bile pigment effect. In addition, we evaluated whether poor bowel preparation might affect the accuracy of lesion recognition by CE trainees in CE-FICE. Our results will help to elucidate the clinical usefulness of CE-FICE.

## Methods

### Patients

Of the 98 patients who underwent CE between February 2010 and March 2011 at Yokohama City University School of Medicine, CE expert selected 12 patients who had various lesions of the small bowel and they were enrolled in this study. All of the 12 patients had undergone CE for determination of the cause of obscure gastrointestinal bleeding. The study protocol was approved by the Yokohama City University Hospital Ethics Committee. Written informed consent was obtained from all the subjects prior to their participation in the study. The patients were instructed to swallow the CE capsule (PillCam SB2; Given Imaging Ltd) with a solution of dimethicone after overnight fasting without any other preparation. The patients were allowed to drink clear liquids at 2 hours and eat light meals at 4 hours after swallowing the capsule. Two CE experts (with experience of reporting more than 150 CE videos) separately read and interpreted the complete CE videos of the 12 patients. To confirm the diagnostic accuracy, intra-observer differences were calculated. If discrepancies were observed, a consensus was reached after the findings were reviewed simultaneously by both the CE experts.

### CE trainees

Four gastroenterology trainees without CE experience (A, B, C, and D) were asked to read and interpret the 12 CE videos. All the trainees had performed at least 500 gastroscopies and 50 colonoscopies. They were given the same introduction to the CE reporting software and given lectures on the typical findings of small bowel lesions (angioectasia and erosions/ulcerations). Prior to this study, they had read one CE video and were given comprehensive feedback by the CE experts.

### FICE technology for CE

**Figure 1:**
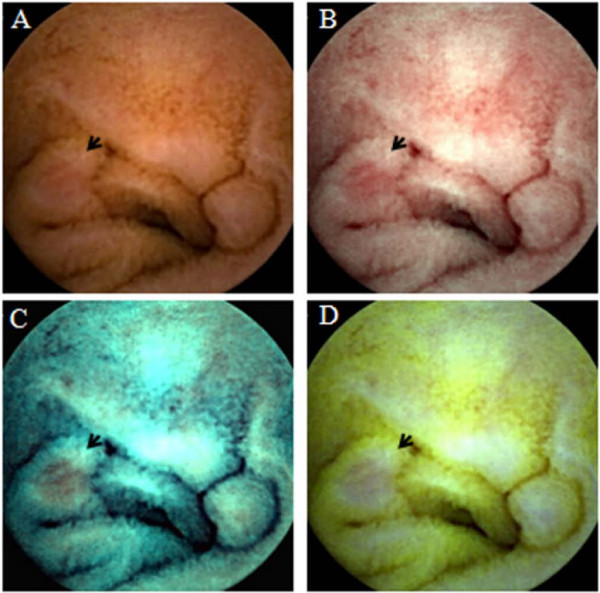
**Example of an erosive lesion.** The lesion is more clearly visualized by CE-FICE. **A**: conventional CE image. **B-D**: CE-FICE images obtained under three different wavelength settings (B: setting 1, C: setting 2. D: setting 3).

The principle of the FICE estimation technology is described elsewhere [19]. In brief, this technology takes an ordinary endoscopic image and arithmetically processes the reflected photons for three different wavelengths (red, green and blue) to reconstitute virtual images. The wavelengths for the FICE settings used in the CE evaluation were as follows; setting 1 (red 595 nm, green 540 nm, blue 535 nm), setting 2 (red 420 nm, green 520 nm, blue 530 nm), setting 3 (red 595 nm, green 570 nm, blue 415 nm) (Figure 1). These three different settings made it possible to flexibly select the most suitable wavelengths required for the evaluation of the capsule videos.

### Study design

**Figure 2:**
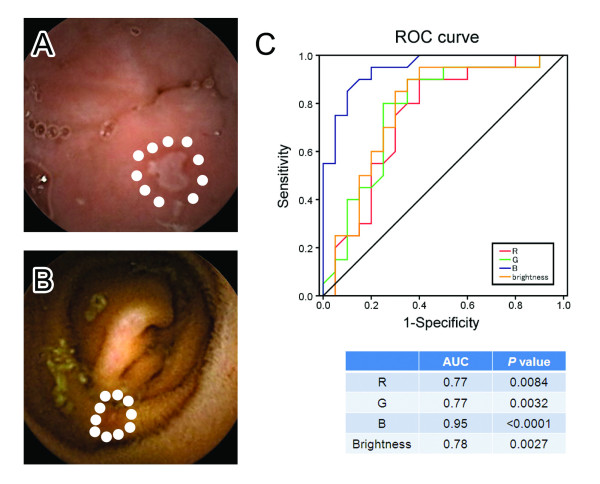
**Definitions of the bile-pigment-positive and bile-pigment-negative condition.** CE experts selected 20 obviously bile-pigment-positive endoscopic images (**A**) and 20 bile-pigment-negative endoscopic images (**B**) that contained small bowel lesions. These images were regarded as golden standard. For each of the images, the RBG (red, blue and green) and brightness (average of ten mucosal points around the small bowel lesions, shown as white dots in this figure) values were calculated. To evaluate the most useful factor for determining whether or not bile pigments are present, a receiver operating characteristic (ROC) analysis was performed (**C**). The curve was obtained by calculating the sensitivity and specificity of the test at every possible cutoff point, and plotting the sensitivity against 1-specificity. The sensitivity was defined as the proportion of bile-pigment-positive images, determined from each of the color values (RBG and brightness value). The specificity was defined as the proportion of bile-pigment-negative images, also determined from the color values. As shown in Table, the results revealed that the B value was the most useful factor to determine the bile pigment condition (area under the curve (AUC): 0.95, *P* < 0.0001).

**Figure 3:**
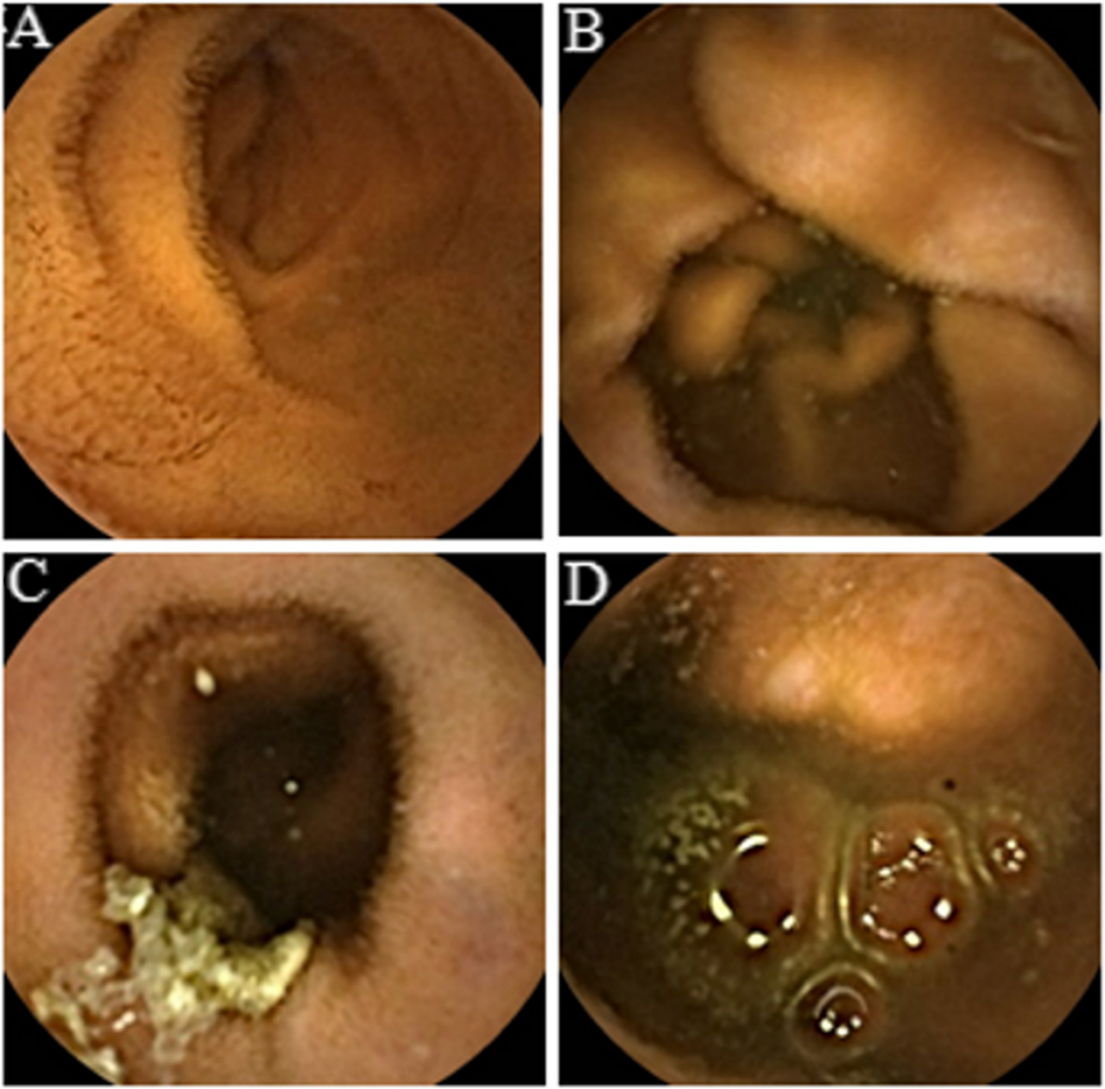
**Definition of bowel visibility.** Visibility was evaluated using a 4-point qualitative evaluation scale: **A** = excellent, visualization of ≥90% of the mucosa, no or minimal fluid, debris and bubbles; **B** = good, visualization of ≥90% of the mucosa, mild fluid, debris and bubbles; **C** = fair, visualization of <90% of the mucosa, moderate fluid, debris and bubbles; **D** = poor, visualization of <80% of the mucosa, excessive fluid, debris and bubbles.

CE experts marked the first duodenum image and the first cecum image to identify the small bowel area. To avoid recall bias, all the trainees were requested to read each of the CE videos of the small bowel area using only one of the visualization protocols (conventional or one of the three different FICE settings) and recorded perceived abnormalities. Each CE video was set to be viewed under all the four settings (for example, case1; trainee A: conventional, B: FICE setting 1, C: FICE setting 2, D: FICE setting 3), therefore, as a result, each CE trainees read 4 videos under the conventional CE setting, 4 under FICE setting 1, 4 under FICE setting 2, and 4 under FICE setting 3. Diagnoses were determined according to samples of the typical findings of small bowel angioectasia and erosions/ulcerations presented by the CE experts. The findings of the CE experts’ were defined as the gold standard. We analyzed the lesion detection rate, as the percentage of lesions, relative to the total number, that were correctly detected by the trainees as compared with that by the experts, for each of the FICE settings. To evaluate whether use of the FICE technology might improve the lesion recognition ability of the CE trainees, the lesion detection rates under each of the FICE settings was compared with that by conventional CE. In addition, CE trainees tend to miss small-bowel lesions in the bile-pigment-positive condition, therefore we also evaluated whether the presence of bile pigments reduced the detectability of the lesions by the CE trainees and whether CE-FICE might contribute to reducing the bile pigment effect. The bile pigment condition was determined by the color values around the small bowel lesions according to the results of the receiver operating characteristic (ROC) analysis (Figure 2). Moreover, we evaluated the frequency of the false-positive findings which were incorrectly thumb-nailed for each visibility grade. False-positive findings were defined as reporting of non-pathological conditions, such as food residues and capillary vessels. According to previous reports [22,23], we scored the visibility using a 4-point qualitative evaluation scale (excellent, good, fair and poor) (Figure 3). Each of the 12 CE videos was divided into four segments of equal length, according to the small-bowel transit time, and landmarks were placed to identify the last 10 minutes of every quartile. CE experts evaluated the visibility of each of the segments of 10 minutes' duration using the 4-point qualitative index.

### Statistical analysis

Continuous data are shown as mean ± SD. ROC analysis was performed to determine whether the bile pigment condition was positive or negative. Differences in the lesion detection rates by CE trainees between conventional CE and CE under each of the FICE settings were calculated by the chi-squared test. Differences in the frequency of false-positive findings between conventional CE and CE using each of the FICE settings according to the status of bowel visibility were calculated by the Mann–Whitney *U* test. Intra-observer differences were calculated by kappa statistics.

## Results

### Small bowel lesions detected by CE experts

CE experts detected 142 lesions of the small bowel, including angioectasia (n = 60) and erosions/ulcerations (n = 82). There was a good level of consistency between the two CE experts’ findings, with a value of 0.66.

### Lesion detection rates by CE trainees

The results are shown in Table 1. Of a total of 60 angioectasias, CE trainees identified 26 by conventional CE, 40 under FICE setting 1, 38 under FICE setting 2, and 31 under FICE setting 3. Of a total of 82 erosions/ulcerations, CE trainees identified 38 by conventional CE, 62 under FICE setting 1, 60 under FICE setting 2, and 20 under FICE setting 3. As compared to conventional CE, CE under FICE settings 1 and 2 significantly improved the detectability of angioectasia (*P* = 0.0017 and *P* = 0.014, respectively) and also erosions/ulcerations (*P* = 0.0012 and *P* = 0.0094, respectively). On the other hand, FICE setting 3 did not allow improved detectability of angioectasia and significantly reduced the detectability of erosions/ulcerations (*P* = 0.015).

**Table 1:** Evaluation of the lesion detection rate by the CE trainees for each FICE setting

	**Lesions detected by the CE experts (%)**	**Conventional**	**Setting 1**	**Setting 2**	**Setting 3**
Angioectasia	60 (100)	26 (43.3)	40 (66.7)^*^	38 (63.3)^*^	31 (51.7)
Erosion/ulceration	82 (100)	38 (46.3)	62 (75.6)^†^	60 (73.2)^†^	20 (24.4)^‡^

^*^Comparison of conventional CE with FICE settings 1 and 2 by the chi-squared test (*P* = 0.0017 and *P* = 0.014, respectively).

^†^Comparison of conventional CE with FICE settings 1 and 2 by the chi-squared test (*P* = 0.0012 and *P* = 0.0094, respectively).

^‡^Comparison of conventional CE with FICE setting 3 by the chi-squared test (*P* = 0.015).

### Definition of the bile pigment condition

The color patterns of the endoscopic images were constructed by the wavelengths of three colors (red, green and blue) and the brightness values. Therefore, we hypothesized that these color values and brightness around the small bowel lesions may contribute to determine of the bile pigment condition. First, we selected 20 obviously bile-pigment-positive endoscopic images and 20 bile-pigment-negative endoscopic images containing small bowel lesions (Figure 2). These images were regarded as golden standard. Subsequently, for each of the images, the RBG (red, blue and green) values and brightness (average of ten mucosal points around the small bowel lesion) were calculated. The RBG values and brightness were assessed with a digital scale in the range of 0–255. To evaluate the factor that might be the most useful for determining the presence of bile pigments, ROC analysis was performed. ROC analysis revealed that the B value was the most useful factor to determine the presence/absence of bile pigments (area under the curve (AUC): 0.95, *P* < 0.0001) (Figure 2). Based on this finding, we set the cutoff value at 71.8 (likelihood ratio: 8.5); thus, endoscopic images with average B values of lower than 71.8 were defined as bile-pigment-positive images. Use of this cutoff value allowed classification of the endoscopic images, even where it was difficult to determine the bile pigment condition subjectively. As a result, the bile pigment condition of the remaining 102 images was determined by this guideline.

### Lesion detection rates by CE trainees according to the bile pigment condition

**Figure 4:**
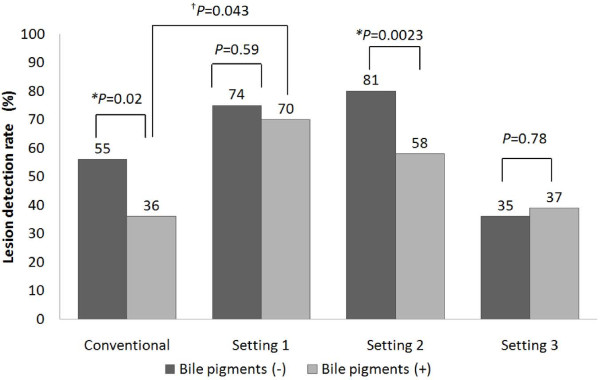
**Evaluation of the lesion detection rate according to the bile pigment condition.** The lesion detection rate under the conventional CE setting and FICE setting 2 were significantly reduced in the presence of bile pigments in the small bowel (*). On the other hand, the lesion detection rate under FICE setting 1 was not affected by the presence of bile pigments. In the bile-pigment-positive condition, the lesion detection rate under FICE setting 1 was significantly higher than that by conventional CE (^†^).

Results are shown in Figure 4. Presence of bile pigments significantly reduced the detectability of small bowel lesions by both conventional CE and CE under FICE settings 2 and 3 (*P* = 0.02 and *P* = 0.0023, respectively). On the other hand, under FICE setting 1, the detectability of these lesions was not affected by the presence of bile pigments (*P* = 0.59). Under the bile-pigment-positive condition, the detectability of small bowel lesions was significantly improved by the use of FICE setting 1 (*P* = 0.043), but not that of setting 2 or 3, as compared with that by the conventional visualization method.

### Number of false-positive findings per visibility score

The results are shown in Table 2. In the presence of poor bowel visibility, the frequency of false-positive findings was significantly increased under FICE settings 2 and 3, as compared with that in conventional CE (*P* = 0.046 and *P* = 0.024, respectively).

**Table 2:** Evaluation of the number of false-positive findings for each visibility score

	**No. of Segments**	**No. of false positive findings/segment**			
		**Conventional**	**Setting 1**	**Setting 2**	**Setting 3**
Visibility score					
excellent	7	0.1 ± 0.4	0.1 ± 0.4	0.3 ± 0.5	0.4 ± 0.5
good	13	0.3 ± 0.1	0.2 ± 0.6	0.2 ± 0.4	0.5 ± 0.7
fair	20	0.6 ± 0.8	0.9 ± 1.0	1.2 ± 1.1	1.7 ± 1.4^*^
poor	8	0.9 ± 0.6	1.6 ± 0.9	2.9 ± 1.9^†^	2.6 ± 1.8^†^

NOTE: Each of the 12 CE videos was divided into four segments of equal length, according to the small bowel transit time, and landmarks were placed to identify the last 10 minutes of every quartile. The CE experts evaluated the visibility of the each segment of 10 minutes' duration using a 4-point qualitative index (excellent, good, fair and poor).

Values are mean ± SD.

^*^Comparison of conventional CE with FICE setting 3 by the Mann–Whitney *U* test (*P* = 0.018).

^†^Comparison of conventional CE with FICE settings 2 and 3 by the Mann–Whitney *U* test (*P* = 0.046 and *P* = 0.024).

## Discussion

Although the lesion recognition ability of the CE trainees enrolled in our study was low, consistent with previous reports [24-27], use of FICE setting 1 or 2 improved the detectability of angioectasia and erosions/ulcerations by the CE trainees. Imagawa et al. reported from a study of CE experts, that CE-FICE improved the detectability of angioectasia, but not that of erosions/ulcerations [28]. Contrary to their results, the detectability of erosions/ulcerations was also improved by CE-FICE in our study. Although CE trainees are often likely to miss small erosions/ulcerations [24,25], FICE enhances the visibility of these lesions, making them easy to recognize by even relatively untrained eyes. Therefore, the detectability of erosions/ulcerations might also be improved by the use of FICE in CE trainees. Angioectasia and erosions/ulcerations represent the major sources of bleeding from the small bowel [29,30], therefore, CE-FICE could be useful for diagnosis of the cause of obscure GI bleeding.

To investigate how CE-FICE might improve the detectability of small bowel lesions, the influence of the presence of bile pigments on the detectability of the small bowel lesions was first evaluated. In our study, the results of the ROC curve analysis revealed that the bile pigment condition was best determined by the B values around the small bowel lesions. As the B values became lower, the images began to look more yellow. Therefore, we concluded that determination of the B value to evaluate the bile pigment condition was reasonable. Our results indicated that while the CE trainees were likely to miss small bowel lesions in the presence of bile pigments, FICE setting 1 appeared to improve the lesion recognizability under this condition. Therefore, use of FICE setting 1 may be clinically meaningful in the bile-pigment-positive condition. Similar to the case for the CE trainees, the presence of bile pigments may affect the detectability of small bowel lesions even by doctors with experience in CE. To verify this contention, however, additional studies are needed.

Our results suggested that CE-FICE requires extra caution under the condition of poor bowel preparation. CE trainees tend to confuse food residues with erosions/ulcerations, and capillary vessels with angioectasia, especially under FICE settings 2 and 3. This indicates that good bowel preparation is preferable for assessing CE videos using FICE.

### Limitations

Although our results indicated the potential clinical usefulness of CE-FICE, there were some limitations of this study. First, we compared the lesion detection rates by the CE trainees, under conventional and three different FICE settings with the findings in the same CE exam previously read by CE experts. The trainees were requested to read each of the CE videos under only any one of the visualization protocols. Although this method might help to avoid recall bias, it dose not provide accurate measurement of the diagnostic accuracy or diagnostic improvement. Second, there is no gold standard at present to confirm whether the area analyzed by RBG image analysis indeed has bile pigments. It is necessarily based on subjective evaluation by the examiner. Third, some lesions reported as false-positives could correspond to new lesions only seen with FICE settings and not necessarily actually false positive. The difficulties in resolving these biases are potential flaws of this study.

## Conclusion

Based on a study of CE trainees, we demonstrated that the detectability of small bowel angioectasia and erosions/ulcerations could be improved by CE-FICE (FICE settings 1 and 2). Moreover, we showed that FICE setting 1 enabled reduction of the bile pigments effect and improved the detectability of small bowel lesions. In addition, our results also indicated that good bowel preparation is important for assessment by CE-FICE.

## Abbreviations

CE, Capsule endoscopy; FICE, Flexible spectral imaging color enhancement.

## Competing interests

The authors have no competing interests to declare.

## Authors’ contributions

ES, HE and HT designed the study. ES wrote the article. KH, EY, HO and TH participated in the critical revision of the manuscript. TM, WT, LT and TU participated in the study as CE trainees and interpreted the CE videos. SK performed the statistical analysis. HE, YH and AN were responsible for the design of the study. All authors read and approved the submission of the final manuscript.

## Authors’ information

ES and HE are physicians specializing in small bowel diseases, and belong to the Gastroenterology division, Yokohama City University School of Medicine, 3–9 Fuku-ura, Kanazawa-ku, Yokohama, 236–0004 Japan.

## Pre-publication history

The pre-publication history for this paper can be accessed here:

http://www.biomedcentral.com/1471-230X/12/83/prepub
